# Efficacy of foam sclerotherapy with polidocanol for the management of oral venous malformations

**DOI:** 10.3892/mi.2024.148

**Published:** 2024-03-26

**Authors:** Tomoki Kato, Yasuhiro Katayama, Shizuko Fukuhara, Takuma Watanabe, Shigeki Yamanaka, Kazumasa Nakao, Naoki Morimoto

**Affiliations:** 1 Department of Oral and Maxillofacial Surgery, Graduate School of Medicine, Kyoto University, Kyoto 606-8507, Japan; 2 Department of Oral and Maxillofacial Surgery, Ama Municipal Hospital, Ama, Aichi 490-1111, Japan; 3 Department of Plastic and Reconstructive Surgery, Graduate School of Medicine, Kyoto University, Kyoto 606-8507, Japan

**Keywords:** oral venous malformations, polidocanol, foam, sclerotherapy, hemangioma

## Abstract

The present aimed to examine the effectiveness of polidocanol-based foam sclerotherapy for oral venous malformations (OVMs). The present study performed a retrospective analysis of patients with OVMs who underwent sclerotherapy using polidocanol. Patients achieving the complete resolution of OVM were categorized as having a complete response (CR), those with a reduction in size from the initial diagnosis were categorized as having a partial response (PR), those with no change in size as stable disease (SD), and those with an increase in size as progressive disease (PD). A total of 16 patients, comprising 4 males and 12 females, underwent treatment with polidocanol foam therapy, covering 22 affected areas. The treatment administered resulted in CR in 6 cases and PR in 10 cases, with no instances of SD or PD. Apart from localized injection site pain or swelling, there were no severe side-effects reported, such as circulatory dynamic changes or skin necrosis. On the whole, these findings underscore the effectiveness of foam sclerotherapy with polidocanol as a viable treatment for venous malformations in the oral and maxillofacial regions.

## Introduction

Hemangiomas and vascular malformations (VMs) in the head and neck regions have traditionally been referred to as ‘hemangiomas.’ Currently, the International Society for the Study of Vascular Anomalies (ISSVA) classifies conventional cavernous hemangiomas as VMs (1). VMs in the head and neck region account for ~40% of all VMs, with an incidence of 1:5,000 to 1:10,000(2). The treatment options for VM include surgery, sclerotherapy, and laser therapy (1). Usually, surgical resection cannot be performed without causing functional or aesthetic impairment and may only be suitable for small localized lesions. Therefore, sclerotherapy has become a valuable alternative to the surgical resection of VMs in the maxillofacial region (3). Compared with conventional liquid sclerosing agents, foam sclerotherapy has been reported to be more effective at low concentrations and in small doses, thereby reducing complications and enabling safe and effective treatment (4). Although there have been some reports (5,6) on the effectiveness of polidocanol sclerotherapy without foam for VMs in the maxillofacial region, to the best of our knowledge, no study to date has evaluated the effectiveness and safety of foam sclerotherapy using only foam polidocanol for VMs in the oral and maxillofacial region. The present study thus aimed to examine its therapeutic effectiveness in patients with oral VMs (OVMs).

## Patients and methods

### Study population and ethical approval

The present study retrospectively examined the medical records of all patients treated between January, 2018 and March, 2023 at Kyoto University Hospital (Kyoto, Japan) who underwent foam sclerotherapy with polidocanol for VMs of the oral and maxillofacial regions. Patients who received foam polidocanol sclerotherapy outside the aforementioned hospital were excluded from the study. Foam sclerotherapy with polidocanol was used when surgical treatment would lead to extensive resection and possible functional or aesthetic impairment or when the patient preferred non-surgical treatment. Data regarding sex, age at the time of the initial visit, and clinical and photographic findings were collected. All patients underwent magnetic resonance imaging (MRI) to assess the extent and distribution of the lesions. The diagnosis of VM was based on history and clinical and MRI findings.

The present study was approved by the Kyoto University Graduate School and Faculty of Medicine Ethics Committee (Approval no. R 3495) and was conducted in accordance with the principles outlined in the Declaration of Helsinki. Informed consent was obtained from all patients and, in the case of minor patients, from their parents through an opt-out option on the website of the department of Oral and Maxillofacial Surgery (https://oms.kuhp.kyoto-u.ac.jp).

### Process of foam sclerotherapy with polidocanol

**Figure 1:**
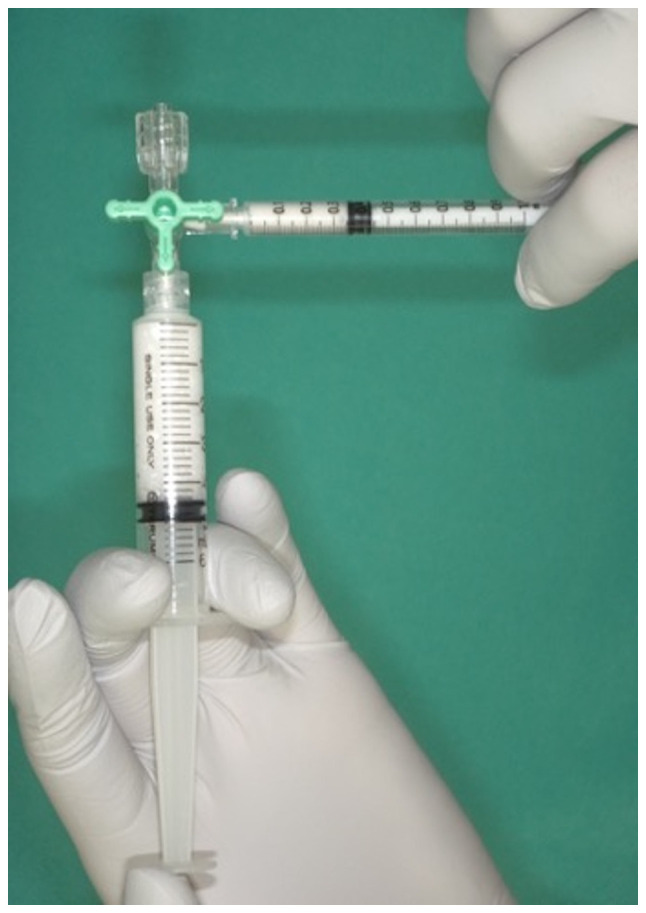
Form making two syringes and a three-way tap. By alternating the plunger, 1 ml polidocanol and 4 ml air are mixed.

The curing material used was 1% polidocanol (Chemical Factory Kreussler & Co. GmbH), obtained by mixing 1 ml polidocanol with air in a 1:4 ratio in two syringes attached to a 3-way stopcock, as previously reported in the study by Tessari *et al* (6) (Fig. 1). The resulting foam polidocanol was then administered via a 22 G needle into the VM. The same site was used during each treatment session, which was spaced at least 2 months apart. This treatment interval was selected in order to allow for an accurate assessment of the disappearance of swelling directly associated with sclerotherapy and to ensure the precise evaluation of the clinical condition of patients. Sclerotherapy was conducted at a maximum dosage of 2 mg/kg and terminated when the lesions completely disappeared upon a visual examination or upon patient satisfaction. The number of sclerotherapy sessions and post-treatment complications were recorded. Complications did not include acute swelling of the treated lesion or transient pain exacerbation following sclerotherapy, as polidocanol typically causes inflammation in and around venous malformations. Patients with transient difficulty opening their mouths following sclerotherapy were also excluded. The treatment response was retrospectively evaluated by two surgeons, one oral surgeon, and one plastic surgeon, using photographs and chart entries in the medical records at follow-up.

### Data analysis

Patients with the complete resolution of the OVM were considered to have a complete response (CR), those with a reduction in size from the initial diagnosis were considered to have a partial response (PR), those with no change in size were considered to have stable disease (SD), and those with an increase in size were considered to have progressive disease (PD).

### Statistical analysis

Characteristics were examined for all participants, and group differences were assessed based on therapy evaluation, with participants categorized into the CR and PR groups. Differences in numerical variables (age and drug frequency) between the subgroups were determined using a Student's t-test. The difference in a nominal variable (sex) between the subgroups was determined using a Fisher's exact test. A two-tailed P-value <0.05 was considered to indicate a statistically significant difference. All statistical analyses were performed using JMP 16.0 statistical software (SAS Institute).

## Results

A total of 16 patients, comprising 4 male and 12 female patients, underwent foam sclerotherapy with polidocanol. The mean age of the patients was 46.8 years (range, 9-80 years). Among the patients, 4 patients had OVMs on multiple sites in the oral and maxillofacial regions, while 12 patients had OVMs on a single site, resulting in a total of 22 sclerotherapy sites. The following sites were observed: A total of eight OVM sites were on the lips, six were on the tongue, six on the buccal mucosa, and one each on the masseter muscle and pharynx. Notably, the VMs were superficial apart from those in the pharynx and masseter area. The average number of polidocanol doses was four (ranging from one to nine doses). The treatment outcome was CR in 6 cases and PR in 10 cases; no patients were found to have SD or PD. Other than pain and swelling at the injection site, no severe side effects, such as circulatory dynamic fluctuations or skin necrosis, were observed in the patients. In addition, no adverse events, such as blood clots, were noted (Table I). Statistically and descriptively, no obvious differences were found in various factors, including age and the site of VM occurrence, by therapy evaluation (Table II).

## Discussion

Hemangiomas are relatively common in the maxillofacial regions. The majority of hemangiomas are considered vascular malformations that do not exhibit neoplastic proliferation of blood vessel-derived cells and cause local dysfunction, bleeding, and aesthetic issues (7). In 1982, Mulliken and Glowacki (8) examined the presence of endothelial cell proliferation in diseases previously considered as hemangiomas and performed a histopathological classification of these diseases. They classified conventional hemangiomas into two groups as follows: i) Those with proliferative and quiescent phases of endothelial cells and ii) those without proliferation of endothelial cells and with a normal cell cycle. Vascular malformations were further subclassified into arteriovenous, capillary, venous, and lymphatic. Currently, the ISSVA classifies ‘hemangiomas’ and ‘vascular malformations’ as separate disease entities (1).

The treatment for VMs in the oral cavity includes surgery, sclerotherapy, and laser therapy (1). Surgical resection can result in functional and aesthetic impairments. By contrast, sclerotherapy is a minimally invasive treatment option for vascular malformations that can be treated without causing functional or aesthetic impairment (3). Sclerosing agents damage vascular endothelial cells, causing thrombosis and subsequent fibrosis (7). Anhydrous ethanol, ethanolamine oleate, and polidocanol are frequently used as sclerosing agents (9). A previous study reported that anhydrous ethanol had the lowest recurrence rate and was the most effective sclerosing agent (10). However, several complications associated with anhydrous ethanol sclerotherapy have been reported, and these include tissue necrosis, peripheral nerve damage, cardiac arrhythmia, and pulmonary embolism (11). Monoethanolamine oleate has excellent thrombogenic potential but needs to be administered under fluoroscopic guidance, as leakage from the lesion can cause hemolysis and acute renal failure (5).

Polidocanol is a non-ionic surfactant that lyses the endothelial layer via absorption into the cell membrane (12). Polidocanol has been reported to be effective in ~90% of OVMs and can be used for sclerotherapy without unique administration methods with a limited number of complications (5). Although polidocanol is associated with relatively fewer complications than other sclerosing agents, Marrocco-Trischitta *et al* (13) reported cases of cardiac arrest when polidocanol was used in pediatric patients, suggesting the need for careful administration when used in children.

In the present study, all patients achieved CR and PR. However, multiple doses are necessary to achieve a sufficient response for extensive lesions. Therefore, multiple treatment cycles required for sclerotherapy with foam polidocanol are a vital drawback to consider. To minimize the number of sclerotherapy and achieve complete disappearance of the lesion, a possible treatment strategy would be to use sclerotherapy to reduce the VM to a size that does not compromise functionality and esthetics, followed by surgical intervention.

Foam sclerotherapy is a type of sclerotherapy wherein a sclerosing agent is mixed with air or carbon dioxide to form a froth. This technique was first used by McAusland (14) in 1939 to treat telangiectasias by shaking the sclerosing agent violently in a rubber stopper bottle to form a froth. In 1944, Orbach (15) reported foam sclerotherapy as an ‘air-block technique’. Compared with conventional liquid sclerosing agents, foam sclerotherapy has been reported to be more effective at low concentrations and in small doses, thus reducing complications and enabling safe and effective treatment (4). The smaller the foam formed, the more effective the treatment (16). According to the method described in the study by Tessari *et al* (6), a 1:4 ratio of hardener solution to air is the optimal mixture, and pumping >20 times does not affect foam formation.

Cabrera *et al* (17) reported that foam sclerotherapy with polidocanol and carbon dioxide was effective in 92% of patients and eliminated VMs in 36% of patients. Yamaki *et al* (18) conducted a randomized controlled trial of liquid sclerotherapy with polidocanol and ethanolamine oleate vs. foam sclerotherapy with polidocanol and ethanolamine oleate. They reported that foam sclerotherapy was significantly more effective than liquid sclerotherapy, and significantly fewer sclerosing agents were used in the former. Sclerotherapy with polidocanol foam effectively achieved CR and PR in patients without any severe complications. However, the present study did not include cases involving infants. Thus, further studies are required to explore the efficacy and safety of polidocanol sclerotherapy in infants.

Given that polidocanol causes inflammatory reactions in and around VMs, MRI imaging was not performed due to concerns that accurate evaluation would not be possible as it would detect not only the reduced venous malformation but also the surrounding inflammatory reactions. Future considerations should focus on refining image evaluation methods.

The limitations of the present study include the small sample size, the lack of pediatric indications and radiological evaluation of lesion size, and the lack of a defined follow-up period. In the future, the authors aim to increase the number of patients and conduct a multicenter collaborative study.

In conclusion, the present study evaluated the efficacy and safety of foam sclerotherapy with polidocanol for VMs in the oral and maxillofacial region, demonstrating it as an effective and safe treatment modality. However, further multicenter collaborative studies are crucial in order to obtain more comprehensive information.

## Figures and Tables

**Table I:** Patients with OVMs treated with foam polidocanol sclerotherapy.

Patient no.	Age, years	Sex	Site	Side	Size (mm)	Dosing frequency (times)	Therapy evaluation
1	11	F	Lip	R	15	8	PR
			Cheek				
2	77	M	Tongue	L	90	9	PR
			Cheek				
			Lip				
3	49	F	Lip	R	12	5	CR
4	71	F	Tongue	R	25	4	CR
5	30	F	Masseter	L	27	3	CR
6	33	F	Lip	R	30	3	CR
7	9	F	Cheek	L	52	5	PR
8	32	F	Lip	R		7	PR
			Tongue				
9	17	F	Lip	R	60	2	PR
10	76	F	Lip	L	5	1	CR
11	78	M	Lip	R	20	4	PR
12	53	M	Tongue	B	35	5	PR
13	54	F	Cheek	L	75	4	PR
			Tongue				
			Pharyngeal				
14	50	F	Cheek	R	12	2	PR
15	80	M	Tongue	L	10	1	CR
16	29	F	Cheek	R	14	1	PR

OVMs, oral venous malformations; F, female; M, male; R, right; L, left; B, both; PR, partial response; CR, complete response.

**Table II:** Differences in parameters according to the therapy evaluation.

	Therapy evaluation		
Parameter	CR, n=6 (37.5%)	PR, n=10 (62.5%)	P-value
Age (years)	56.5±22.2	41.0±25.4	0.236
Sex (n, %)			NS
Male	1 (25.0)	3 (75.0)	
Female	5 (41.7)	7 (58.3)	
Drug frequency (times)	2.8±1.6	4.7±2.7	0.145
Side (n, %)			
Right	3 (30.0)	7 (70.0)	
Left	3 (42.9)	4 (57.1)	
Both	0 (0.0)	1 (100.0)	
Site (n, %)			
Lip	3 (37.5)	5 (62.5)	
Buccal mucosa	0 (0.0)	5 (100.0)	
Tongue	2 (28.6)	5 (71.4)	
Masseter	1 (100.0)	0 (0.0)	
Pharyngeal	0 (0.0)	1 (100.0)	

Values are presented as the mean ± standard deviation or number (frequency, %). CR, complete response; PR, partial response; NS, not significant. Statistical significance was assessed using the Student's t-test and the Fisher's exact test.

## Data Availability

The datasets used and analyzed in the current study are available from the corresponding author upon reasonable request.
